# Dielectric collapse at the LaAlO_3_/SrTiO_3_ (001) heterointerface under applied electric field

**DOI:** 10.1038/s41598-017-09920-9

**Published:** 2017-08-25

**Authors:** M. Minohara, Y. Hikita, C. Bell, H. Inoue, M. Hosoda, H. K. Sato, H. Kumigashira, M. Oshima, E. Ikenaga, H. Y. Hwang

**Affiliations:** 1 Stanford Institute for Materials and Energy Sciences, SLAC National Accelerator Laboratory, Menlo Park, California, 94025 USA; 2 Geballe Laboratory for Advanced Materials, Department of Applied Physics, Stanford University, Stanford, California, 94305 USA; 3 Department of Advanced Materials Science, The University of Tokyo, Kashiwa, Chiba, 277-8561 Japan; 4 Photon Factory, Institute of Materials Structure Science (IMSS), High Energy Accelerator Research Organization (KEK), Tsukuba, Ibaraki, 305-0801 Japan; 5 Department of Applied Chemistry, The University of Tokyo, Bunkyo-ku, Tokyo, 113-8656 Japan; 6 Japan Synchrotron Radiation Research Institute (JASRI), SPring-8, Sayo, Hyogo 679-5198 Japan; 7 Photon Factory, Institute of Materials Structure Science (IMSS), Present Address: High Energy Accelerator Research Organization (KEK), Tsukuba, Ibaraki 305-0801 Japan; 8 H. H. Wills Physics Laboratory, Present Address: University of Bristol, Tyndall Avenue, Bristol BS8 1TL UK

## Abstract

The fascinating interfacial transport properties at the LaAlO_3_/SrTiO_3_ heterointerface have led to intense investigations of this oxide system. Exploiting the large dielectric constant of SrTiO_3_ at low temperatures, tunability in the interfacial conductivity over a wide range has been demonstrated using a back-gate device geometry. In order to understand the effect of back-gating, it is crucial to assess the interface band structure and its evolution with external bias. In this study, we report measurements of the gate-bias dependent interface band alignment, especially the confining potential profile, at the conducting LaAlO_3_/SrTiO_3_ (001) heterointerface using soft and hard x-ray photoemission spectroscopy in conjunction with detailed model simulations. Depth-profiling analysis incorporating the electric field dependent dielectric constant in SrTiO_3_ reveals that a significant potential drop on the SrTiO_3_ side of the interface occurs within ~2 nm of the interface under negative gate-bias. These results demonstrate gate control of the collapse of the dielectric permittivity at the interface, and explain the dramatic loss of electron mobility with back-gate depletion.

## Introduction

Since the discovery of a variety of interfacial electronic states between insulating and non-magnetic (001)-oriented LaAlO_3_ and SrTiO_3_, such as high-mobility metallic states^1^, superconductivity^2, 3^, and magnetism^4–7^, the origin of these properties has been widely discussed^8, 9^. Given the robust insulating character of LaAlO_3_, it is generally understood that the electron gas forms on the SrTiO_3_ side of the interface^10–17^. Indeed, an *in situ* photoemission spectroscopy (PES) study revealed downward band bending toward the interface in the SrTiO_3_^17^. Notable in this system is the dramatic tunability of the interfacial conductivity using external electric fields, attracting considerable attention for fundamental studies, as well as device applications^18, 19^. The application of a back-gate voltage *V*_g_ tunes multiple parameters in the system simultaneously, including the superconducting transition temperature, the carrier density, the Hall mobility and the confining electric field^3, 18, 20–23^. In order to understand how these changes are inter-related, especially the dramatic loss of Hall mobility with back-gate depletion^20^, knowledge of the band alignment and potential profile changes with gating is essential.

**Figure 1:**
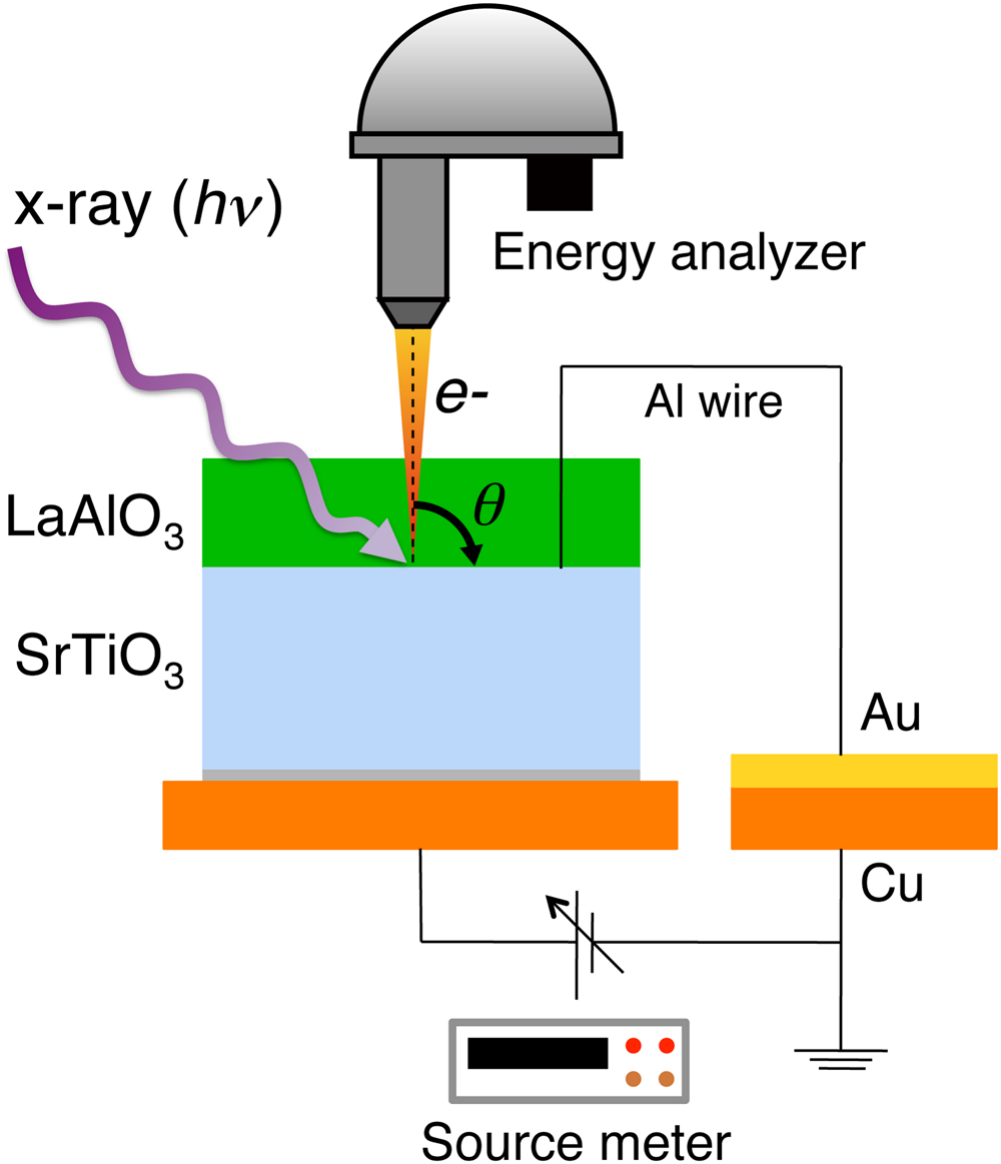
Experimental setup for measurements of PES spectra for LaAlO_3_/SrTiO_3_ heterostructures with applied electric field at the back of the SrTiO_3_. Ground contact is made to the electron gas at the heterointerface using Al wire bonding. The photoelectron emission angle (*θ*) is defined as the angle from the surface normal.

In this study, we analyze the depth profile of the potential on the SrTiO_3_ side of LaAlO_3_/SrTiO_3_ (001) heterojunctions using synchrotron radiation PES for various *V*_g_. A schematic of the experimental setup is shown in Fig. 1. Depth resolution was achieved by varying the energy of the synchrotron-radiation light source - both soft x-ray PES (SX-PES) and hard x-ray PES (HAX-PES) were utilized - combined with precise tuning of the incident and emission angles^24^. Analysis of the SX-PES and HAX-PES core-level spectra with negative *V*_g_ reveal an abrupt downward shift of the potential, narrowing the electron confinement to within ~2 nm of the LaAlO_3_/SrTiO_3_ interface. These results explain why back-gate depletion modulates the mobility far more strongly than the carrier density, and suggests this is a generic feature of nonlinear dielectrics that can be utilized in device structures.

## Results

### Photoemission core-level spectroscopy of LaAlO_3_/SrTiO_3_

**Figure 2:**
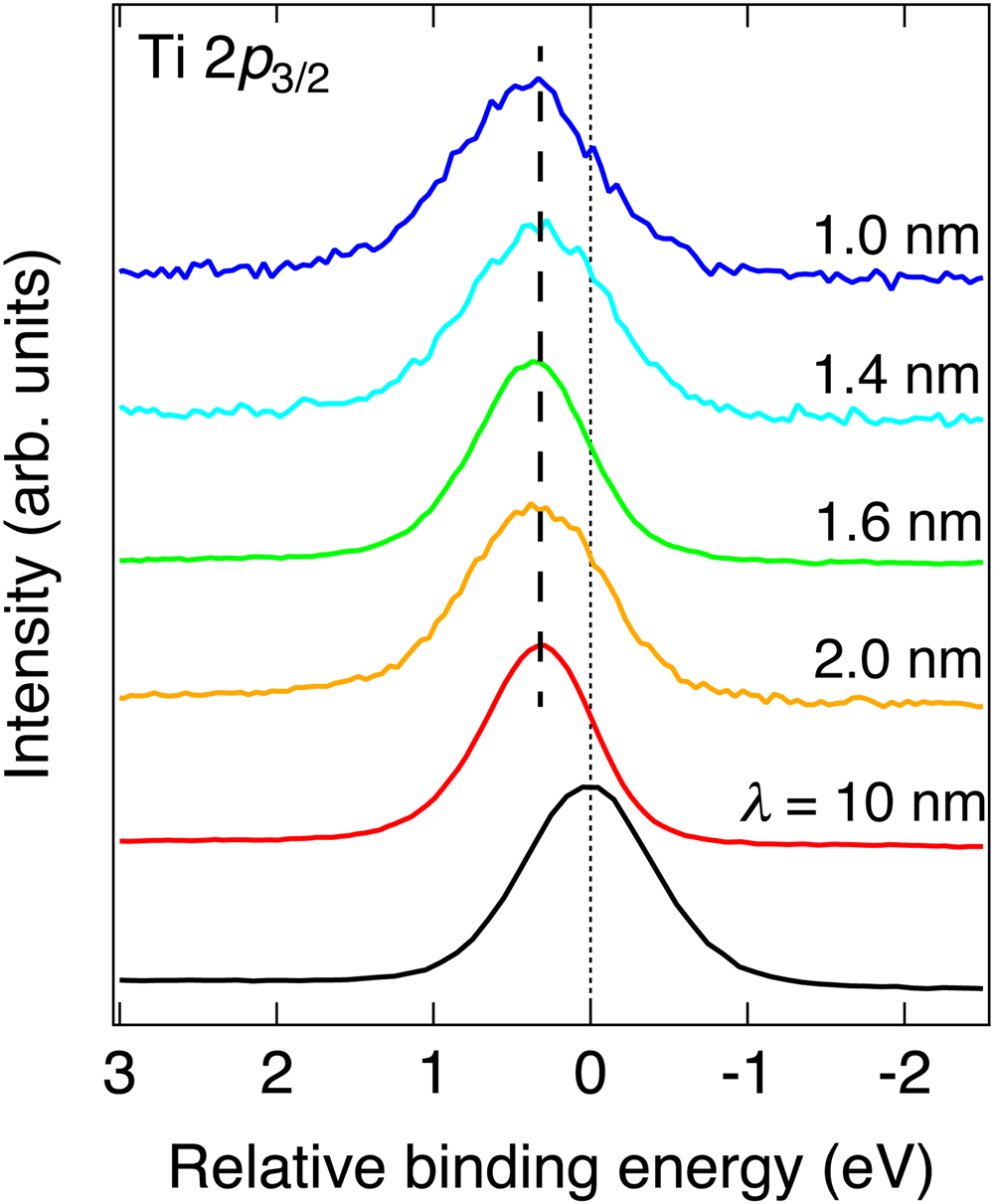
Ti 2*p* core-level spectra of LaAlO_3_/SrTiO_3_ heterostructures without bias gate voltage. The relative binding energy is given with respect to the Ti 2*p* core-level spectra of a bare SrTiO_3_ substrate as reference (black line). *λ* is the probing depth controlled by photon energy and emission angle; *λ* = 1.6 and 10 nm are obtained by HAX-PES (*hν* = 7.9 keV) with *θ* = 80 and 0 degrees, respectively, while others (*λ* = 2.0, 1.4 and 1.0 nm) are SX-PES (*hν* = 1.2 keV) results with varied angles at *θ* = 0, 45, and 60 degrees, respectively.

The measured Ti 2*p* core-level spectra of the LaAlO_3_/SrTiO_3_ heterostructures at *V*_g_ = 0 V and a bare SrTiO_3_ (001) substrate are shown in Fig. 2. The characteristic probing depth *λ*, based on the theoretical study of Tanuma *et al*.^25^, was tuned by changing the irradiation photon energy (*hν*) and the photoelectron emission angle (*θ*) with respect to the surface normal. *hν* was 1.2 keV (*λ* = 2.0 nm for *θ* = 0 degree) and 7.9 keV (*λ* = 10 nm for *θ* = 0 degree), *θ* was varied from 0 to 80 degrees (also see Fig. 1). The Ti 2*p* core-level spectra from the LaAlO_3_/SrTiO_3_ sample are shifted towards more positive relative binding energy from that of the bare SrTiO_3_ substrate with no observable Ti^3+^ component, as previously found^17^. As the probing depth becomes shallower (*λ* < 10 nm), no significant peak shift or broadening characteristic to potential variation, was observed within the experimental resolution of 50 meV. These results together with the gate-tunable transport properties^20^ imply that the potential profile varies on the scale of 10 nm or more in the SrTiO_3_ from the LaAlO_3_/SrTiO_3_ heterointerface. By assuming that all carriers detected from Hall effect under no applied bias (*n*_2D_ ~2 × 10^13^ cm^−2^) reside within 0.4 nm of SrTiO_3_ surface and fully contribute to the Ti^3+^ signal, the estimated total volume of Ti^3+^ is still below its practical detection limit of 2~3 at. %. Moreover, the possible carrier distribution, which is interrelated with the potential profile, experimentally prohibits the observation of any Ti^3+^ states present.

**Figure 3:**
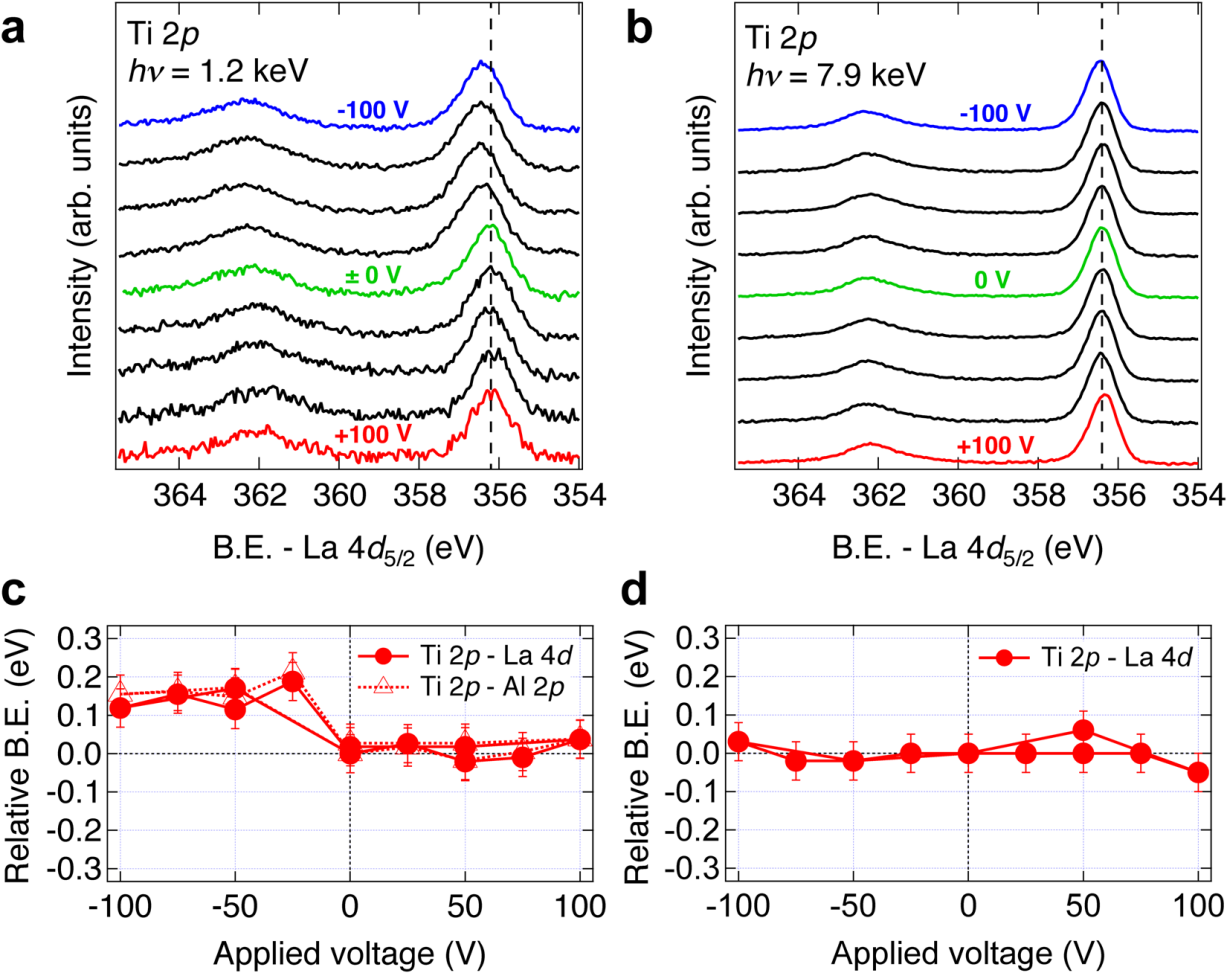
Ti 2*p* core-level spectra of LaAlO_3_/SrTiO_3_ heterostructures measured using (**a**) SX-PES (*hν* = 1.2 keV, *θ* = 0 degree) and (**b**) HAX-PES (*hν* = 7.9 keV, *θ* = 0 degree) with applied gate voltage. Plots of relative binding energy (B.E.) shift between (**c**) Ti 2*p* and La 4*d* core-level spectra (filled circles), Ti 2*p* and Al 2*p* (open triangles) for SX-PES data, and (**d**) Ti 2*p* and La 4*d* core level spectra (filled circles) from HAX-PES data. The error bars in (**c**,**d**) were obtained from the accuracy of the peak fitting. The two sets of data points correspond to the forward and backward voltage sweeps to evaluate a possible hysteresis.

Figure 3 show the measured Ti 2*p* core-levels using (a) SX-PES and (b) HAX-PES with −100 V ≤ *V*_g_ ≤ 100 V. For all *V*_g_, the gate leakage current through the SrTiO_3_ during the PES measurements was less than 10 nA. The binding energy of the SrTiO_3_ core-level spectra were normalized to those of the LaAlO_3_ spectra, which minimizes possible artifacts from the PES measurements, such as fluctuations of the photon energy. For negative *V*_g_, the Ti 2*p* core-level first shifts to more positive relative binding energy from *V*_g_ = 0 V before saturating at +0.1~+0.2 eV in the case of SX-PES, while it remains unchanged for the HAX-PES. Both SX-PES and HAX-PES measurements show no Ti 2*p* core-level shift for positive *V*_g_. Similar results were also obtained in the Sr 3*d* core-level measurements. The change of the Ti 2*p* core-level peak position are plotted as a function of *V*_g_ in Fig. 3(c) and (d), showing a total energy shift ~+0.15 eV for *V*_g_ < 0 V for the SX-PES without any hysteresis.

Qualitatively we can examine these results by considering the simultaneous tuning of the sheet carrier density and the potential profile due to the application of negative *V*_g_. Considering the SrTiO_3_ substrate as a capacitor dielectric between the back gate contact and the interface conducting layer, *V*_g_ < 0 V corresponds to depletion of carriers at the interface resulting in a downward shift of the Fermi energy (*E*_F_) towards the conduction band bottom. At the same time however the band-bending is enhanced by the gating. This shifts the center of the electron distribution closer to the interface. For fixed sheet carrier density, *n*_2D_, the increased confinement shifts *E*_F_ upwards in energy, opposite to the effect of depletion. Since the SX-PES Ti 2*p* core-level shifts to *higher* binding energy for *V*_g_ < 0 V the band-bending induced upshift in *E*_F_ is dominant. Indeed the dielectric constant of SrTiO_3_ at room temperature *ε*_r_(*T* = 300 K)~350, gives a total carrier density change of ~2 × 10^10^ cm^−2^ for *V*_g_ = −50 V. This is just 0.14% of the measured total Hall sheet carrier density in this sample^20^. The magnitude of the energy shift clearly depends on the probing depth of the PES measurement compared to the characteristic length scale of the confinement potential narrowing. Hence the lack of an observable shift in the HAX-PES data suggests that the most significant changes in the confining potential occur in the topmost layers of the SrTiO_3_.

### Self-consistent simulation for the core-level spectra and potential profile

In order to quantitatively analyze these scenarios, we simulated the potential profile in SrTiO_3_ as a function of the depth (*z*) from the interface based on a modified Thomas-Fermi screening model, using the PES data to constrain the results. The scheme of self-consistent calculation is as follows: for a trial potential Φ(*z*), the carrier density profile *n*(*z*) is calculated using

1 $$n(z)={\int }_{{\epsilon }=0}^{\infty }D({\epsilon })f({\epsilon })d{\epsilon },$$

where *D*(${\epsilon }$) is the density of states at electron energy ${\epsilon }$ from the conduction band minimum which is calculated via $D({\epsilon })={(2{m}^{\ast }/{\hslash }^{2})}^{3/2}{\epsilon }{(z)}^{1/2}/2{\pi }^{2}$, where ${m}^{\ast }$ is the effective mass of SrTiO_3_, $\hslash$ is Plank’s constant divided by 2*π*, and $\,{\epsilon }(z)$ is based on Φ(*z*)^16^, and *f*(${\epsilon }$) is the Fermi-Dirac function. Following ref. 16, we use the three-dimensional form of the density of states. The potential is obtained by solving Poisson’s equation

2 $$\frac{\partial }{\partial z}({\varepsilon }_{0}{\varepsilon }_{r}({\mathbb{E}})\frac{\partial {\rm{\Phi }}(z)}{\partial z})=-{e}_{0}n(z).$$

*ε*_0_ is the vacuum permittivity, *ε*_r_(${\mathbb{E}}$) is the electric field (${\mathbb{E}}$) dependent relative permittivity of bulk SrTiO_3_, and *e*_0_ is the elemental charge. Here, we assumed other sources of charge, e.g. holes, extrinsic donors and acceptors, are negligible. Equations (1) and (2) were solved self-consistently with a convergence criterion of 0.01% throughout the whole depth region. For each *V*_g_, the two boundary conditions used are Φ(*z* = 0) and $\frac{\partial {\rm{\Phi }}(z)}{\partial z}|\,$_*z=∞*_. The former is equal to the energy shift of the Ti 2*p* core-level peak position in the SX-PES spectra at the appropriate *V*_g_^24^, and the latter is 0 V/m and 1 × 10^5^ V/m, for *V*_g_ = 0 and −50 V respectively, given the 0.5 mm SrTiO_3_ substrate thickness. In order to compare the experimentally obtained spectra with our model, we introduce an evaluation function known as the core-level intensity spectrum *I*(*E*). This is generally computed according to

$$I(E)\propto {\sum }_{z=0}^{\infty }\exp (-z/\lambda \,\cos \,\theta )C[E-{E}_{0}-{\rm{\Phi }}(z)],$$

where *C*(*E*) represents a core-level spectrum described by a Voigt function peaked at *E*_0_, which in this case we set to zero as we are interested only in the relative change in the spectrum at different *V*_g_, not in the absolute value of *E*_0_^24^. The final best fit result is determined by minimizing the sum of the squares of the difference between the measured and calculated forms of the *I*(*E*) for both the SX-PES and HAX-PES measurements.

**Figure 4:**
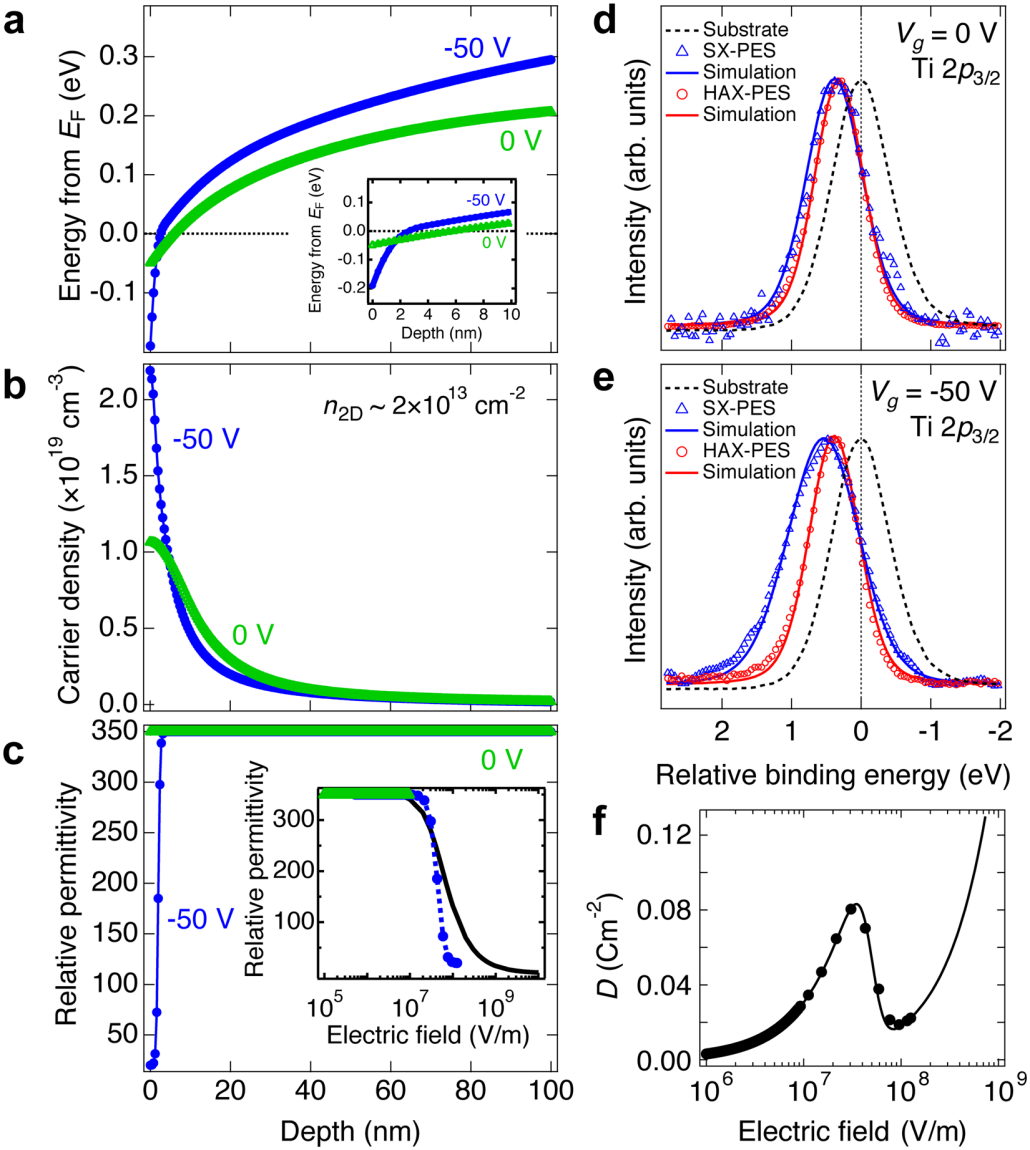
The calculated self-consistent potential and carrier density profiles. (**a**) Simulated potential depth profiles of the electron gas, for *V*_g_ = 0 and −50 V. Inset of Fig. 4(a) shows the magnification around the interface. (**b**) Self-consistent carrier profile and (**c**) the resultant *ε*_r_($$z)$$ for *V*_g_ = 0 and −50 V. The inset of Fig. 4(c) shows the comparison between the extracted form of *ε*_r_($${\mathbb{E}}$$) (symbols and dashed line) and the reported *ε*_r_($${\mathbb{E}}$$) (solid line)^27^. Measured and simulated SX-PES and HAX-PES Ti 2*p* core-level spectra for (**d**) *V*_g_ = 0 V, and (**e**) *V*_g_ = −50 V. Open circles are the experimental data, and solid lines are the best-fit simulations. Dashed lines correspond to the Ti 2*p* core-level spectrum of a bare SrTiO_3_ substrate. (**f**) The calculated electric displacement field from the inset of Fig. 4(c). Solid line is calculated by fitting the extracted form of *ε*_r_($${\mathbb{E}}$$) in the inset of Fig. 4(c) using a sigmoid function.

The electric field (${\mathbb{E}}$) dependent local nonlinear permittivity of SrTiO_3_, *ε*_r_(${\mathbb{E}}$, *z*), must be considered as discussed in previous reports^16, 20, 26^. First we attempted to utilize the reported form of *ε*_r_(${\mathbb{E}}$) in our simulation (Fig. 4(c) inset)^27^. However when *n*_2D_ was constrained to the measured value from Hall effect^20^, satisfactory agreement with the PES spectra was not possible. Even allowing *n*_2D_ to be a free parameter (allowing for the possibility of localized charge not contributing to the Hall measurement), no value could reasonably fit the spectra and other features such as the core-level shift in Fig. 3(c) (see supplementary materials). The key point is that the reported bulk form of *ε*_r_(${\mathbb{E}}$) did not accurately reproduce the nonlinearity of the dielectric permittivity inferred from the data.

As an alternative, we then assumed a smoothly varying dielectric constant as a function of distance from the interface *ε*_r_(*z*) for *V*_g_ = 0 and −50 V, based on a simple sigmoid function, which was used as a physically reasonable qualitative form. The self-consistently solved potential and carrier density profiles are shown in Fig. 4(a) and (b). Here the errors of the simulated parameters were estimated with a threshold of 3% increase in the total squared error from the minimum value, except for the error in Φ(0) which was directly taken from the experimental error in the PES spectra. The comparison between the experimental spectra (open symbols) and the best-fit simulated spectra (solid lines) from the obtained potential profiles are shown in Fig. 4(d) and (e). Good agreement is observed for both the SX-PES and HAX-PES data. The main panel of Fig. 4(c) shows the best fit *ε*_r_(*z*) for *V*_g_ = 0 and −50 V in these simulations, and the insets of Fig. 4(c) shows the extracted *ε*_r_(${\mathbb{E}}$).

From these simulations, close to the interface, we find a relatively abrupt potential shift of ~0.15 eV inside the SrTiO_3_ for *V*_g_ = −50 V compared to *V*_g_ = 0 V. As shown in the inset of Fig. 4(a), the potential crosses *E*_F_ around *z* = 6 nm and 2 nm for *V*_g_ = 0 and −50 V, the former is in good agreement with previous experimental and theoretical estimates of the electron gas thickness being < 10 nm^13, 15, 16, 28, 29^.

## Discussion

Experimentally a significant change of the potential around the interface occurred only for *V*_g_ < 0 V, and not *V*_g_ > 0 V. Based on these calculation results, we can explain this asymmetry by considering the magnitude of ${\mathbb{E}}$ around *z* = 0. For *V*_g_ = 0 V, ${\mathbb{E}}$ is not large enough to significantly reduce *ε*_r_, and positive *V*_g_ only tends to decrease the interfacial electric field. The resultant changes in the band-bending and position of *E*_F_ are therefore relatively small and below the PES resolution. This is in stark contrast to the case of *V*_g_ < 0 V, where ${\mathbb{E}}(z=0)$ is large enough to reduce *ε*_r_, which self-consistently enhances the confinement leading to significant changes in the potential which are measurable by SX-PES.

Quantitatively, the collapse of *ε*_r_ with ${\mathbb{E}}$ obtained in the analysis above is more rapid than reported for non-doped SrTiO_3_^16, 30–32^ and Nb doped SrTiO_3_^27^; the latter being somewhat more strongly affected for a given ${\mathbb{E}}$ than the former one. This has important implications for calculations of electron accumulation layers in any SrTiO_3_–based heterostructure. We note that our self-consistent approach is the same as the one of Copie *et al*., who utilized the literature form of *ε*_r_(${\mathbb{E}}$)^16^. The *ε*_r_(${\mathbb{E}}$) relationship reported by Yamamoto *et al*.^27^ has also been successfully used to model the depletion layer in metal/SrTiO_3_ Schottky junctions^33^. An important difference between the Schottky depletion layer and the LaAlO_3_/SrTiO_3_ is the existence of free electrons in the latter, which can screen applied electric fields in addition to the lattice polarization which is the only possibility in the former. Indeed, a recent theoretical study has noted the interplay between electron density changes and lattice polarization^34^. In order to clarify these points, direct microscopic investigations of the lattice polarization with gate voltage are essential. Noting the large changes in potential over just a few lattice parameters, the failure of the prior experimental measurements of *ε*_r_(${\mathbb{E}}$) to capture our data likely reflect the need to explicitly consider *ε*_r_ on short length scales, and include nonlocal effects^35, 36^. In this sense, our model employing *ε*_*r*_(z) is one of the approximations implicitly incorporating the effect of *k*-dependence of *ε*_*r*_. Despite the flexibility in the functional form of *ε*_*r*_, an abrupt drop in the potential at the interface is an essential feature required to reproduce our spectroscopic results.

Finally we note the intriguing point that when the electric displacement field *D*(${\mathbb{E}}$) is calculated using the new functional form of *ε*_r_(${\mathbb{E}}$), ${\mathbb{E}}$ is multi-valued for 0.02 ≤ *D* ≤ 0.08 Cm^−2^, as shown in Fig. 4(f). Although the underlying physics and effects of such an electrostatic instability are currently not clear, it is possible that the presence of multiple metastable dielectric states in SrTiO_3_ close to the interface could induce local structural phase transitions^37^ and associated effects in resistive switching properties^38^, in addition to creating an unstable potential profile at the interface. The collapse of *ε*_r_ around the interface, simultaneously enhancing the electron confinement and impurity scattering, especially at low temperatures, explains the substantial decrease in the mobility^20^ and enhanced localization^37^ that has been previously observed for back-gating. The strong contrast with top-gating^39^ suggests that this nonlinear dielectric response provides new device switching approaches in oxide heterostructures.

## Methods

The LaAlO_3_/SrTiO_3_ was fabricated on TiO_2_-terminated SrTiO_3_ (001) substrates by pulsed laser deposition as described elsewhere^20^. During LaAlO_3_ depositions, the substrate was kept at a temperature of 800 °C, and the ambient oxygen pressure was maintained at 1 × 10^−5^ Torr. The LaAlO_3_ thickness is 10 unit cells (~4 nm), which is the identical sample used in our previous transport study using back-gate^20^. SX-PES and HAX-PES synchrotron radiation measurements were carried out under applied *V*_g_ at beamline BL2C of the Photon Factory, KEK, Japan and beamline BL47XU of SPring-8, Japan, respectively. The SX-PES and HAX-PES spectra were recorded using a Scienta SES-2002 electron energy analyzer, and a Scienta R-4000 electron energy analyzer, respectively. All PES measurements were performed at room temperature. A schematic of the experimental setup is shown in Fig. 1. *V*_g_ was applied from the back of the 0.5 mm thick SrTiO_3_ substrate during the PES measurements, with the LaAlO_3_/SrTiO_3_ interface grounded using Al wire bonding via a gold-coated copper plate. The back of the SrTiO_3_ substrate was electrically contacted to this copper plate using silver epoxy.

## Electronic supplementary material

Supplementary Info
