# Supplementary material for: Dielectric collapse at the LaAlO3/SrTiO3 (001) heterointerface under applied electric field
Source: Sci Rep. 2017 Aug 25;7:9516. doi: 10.1038/s41598-017-09920-9 (PMC5573322; doi:10.1038/s41598-017-09920-9)
Supplement: Supplementary file 1 — Supplementary Info [file 41598_2017_9920_MOESM1_ESM.pdf]

Supplementary Materials for

Dielectric collapse at the $LaAlO_3/SrTiO_3$ (001) heterointerface under applied electric field

M. Minohara, Y. Hikita, C. Bell, H. Inoue, M. Hosoda, H. K. Sato, H. Kumigashira, M. Oshima, E. Ikenaga, and H. Y. Hwang

Author to whom correspondence should be addressed; Electronic mail: minohara@post.kek.jp

This PDF file includes:

Fig. S1 and S2

Potential profiling simulation based on reported nonlinear permittivity

In order to achieve a full quantitative simulation of the gating dependence of the interface, the local nonlinear permittivity $\varepsilon_r(\mathbb{E}, z)$ of $SrTiO_3$ must be considered. Therefore, we initially tried to utilize the reported $\varepsilon_r(\mathbb{E})$ in our simulation [S1]. Figure S1 shows the potential profiling analysis for $LaAlO_3/SrTiO_3$ corresponding to the measured sheet carrier density of $n_{2D} \sim 2\times10^{13}$ cm^{-2} [S2].

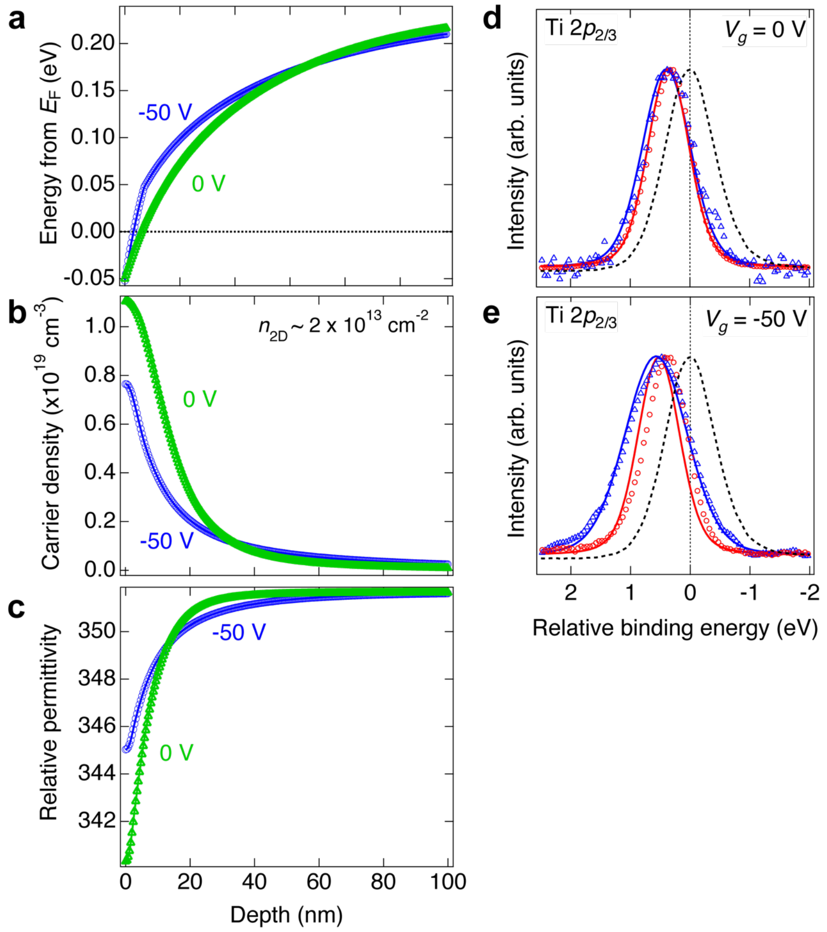

Fig. S1 Self-consistently solved (a) potential profile, (b) carrier profile, and (c) permittivity profile in depth for V_g = 0 (green) and -50 V (blue) corresponding to the measured sheet carrier density. The Ti 2*p* core-level spectra of buried $SrTiO_3$ substrate obtained by SX-PES and HAX-PES measurements (blue and red, respectively) under (d) V_g = 0 V, and (e) -50 V, respectively. Open circles are the experimental results, and solid lines are the simulated results. Black dashed line corresponds to the Ti 2*p* core-level spectra of a bare $SrTiO_3$ substrate.

In this condition, the potential profile cannot explain the measured core-level spectra for V_g = -50 V as shown by the poor quality of the fit in Fig. S1(e). In addition, Fig. S1(a) does not show any energy shift at depth = 0 nm, suggesting no core-level

shifts, while experimentally we clearly observed the core-level shift with an applied negative gate voltage as shown in Fig. 3. Allowing n_{2D} to vary did not resolve these issues, which motivated the use of a sigmoid function for $\varepsilon_r(z)$ as discussed in the main text.

On the effect of photovoltages due to x-ray irradiation during XPS measurements

Because $SrTiO_3$ is a wide band gap semiconductor, there is a possibility of generating photovoltages due to the x-ray irradiation. Here the important point to note is that the penetration depth of the x-rays used for the measurements is in the range of 100s of microns to mm. Since the generated photoelectrons will occur deep inside the $SrTiO_3$ substrate, we would expect a drastic decrease in the resistance of the substrate if this effect was significant. However, as seen from Fig. S2 the current-voltage characteristics measured across the substrate during the XPS measurements, there is no experimentally significant difference especially for negative biases. This result indicates that we can neglect photovoltages during the XPS measurements.

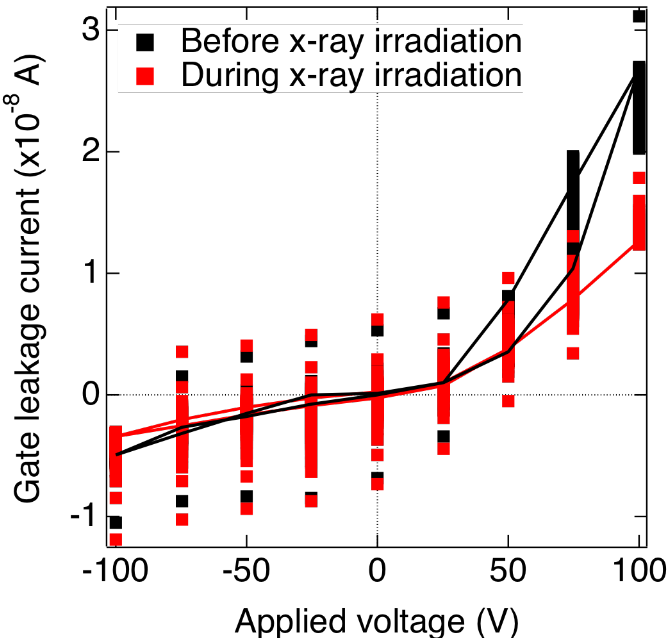

Fig. S2 Current-voltage characteristics from the $LaAlO_3/SrTiO_3$ interface across the $SrTiO_3$ substrate before x-ray irradiation (black) and during XPS measurements (red).

References

S1. Yamamoto, T., Suzuki, S., Suzuki, H., Kawaguchi, K., Takahashi, K. & Yoshisato, Y. Effect of the field dependent permittivity and interfacial layer on $Ba_{1-x}K_xBiO_3$/Nb-doped $SrTiO_3$ Schottky junctions. *Jpn. J. Appl. Phys.* **36**, L390-L393 (1997).

S2. Bell, C., Harashima, S., Kozuka, Y., Kim, M., Kim, B. G., Hikita, Y. & Hwang, H. Y. Dominant mobility modulation by the electric field effect at the $LaAlO_3$/$SrTiO_3$ interface. *Phys. Rev. Lett.* **103**, 226802 (2009).
